# Don´t set us aside!Experiences of families of people with BPD who have access to Brief admission:a phenomenological perspective

**DOI:** 10.1080/17482631.2022.2152943

**Published:** 2022-12-07

**Authors:** Sally Hultsjö, Åsa Appelfeldt, Rikard Wärdig, Jessica Cederqvist

**Affiliations:** a Department of Psychiatry, Ryhov County Hospital. Jönköping, Sweden and Department of Health, Medicine and Caring Sciences. Division of Nursing and Reproductive Health, Linköping University, Linköping, Sweden; b Department of Health, Medicine and Caring Sciences. Division of Nursing and Reproductive Health, Linköping University, Linköping, Sweden

**Keywords:** Brief-admission, experiences, family, health, nursing, phenomenology

## Abstract

**Aim:**

To highlight the experiences of family members of people with borderline personality disorder (BPD) and self-harming behaviour who have access to brief admission

**Methods:**

To understand the families lived experience a phenomenological lifeworld perspective was adopted to this study. Twelve in-depht interviews were performed in November and December 2021 with family members of people with BPD and self-harming behaviour who have accessed BA. The phenomenological life-world perspective guided the analysis.

**Results:**

Families’ life-world was characterized by anxiety and constant protection of their loved one. They live with constant fear of how their loved ones are feeling and whether they will injure themselves. When access to BA was available this gave hope and provided conditions for families to maintain everyday routines and also enhanced relationships among family members. When families’ loved ones were denied BA, they felt betrayed which contributed to negative feelings towards the medical profession, and the families lost confidence in psychiatry.

**Conclusion:**

By interviewing families of people with BPD and self-harming behaviour who had access to BA, it emerged they possess valuable knowledge. BA can be developed if the needs of families are taken into consideration, and if families are given the opportunity to share emotions and the high burden of responsibility with staff or families in similar situations. If health care staff gives family members a more central role in care and makes their shared life-world visible it could thereby hopefully increase well-being and benefits for the whole family

## Introduction

The worldwide lifetime prevalence of borderline personality disorder (BPD) ranges from 1% to 5.9% (Gunderson et al., 2018). BPD is a complex and challenging mental health condition for the person with BPD as well as the family members who support them (Lawn & McMahon, 2015). Among families of people with BPD, psychological suffering is high and the burden is experienced as significant (Kay et al., 2018). Several different factors contribute to this: BPD and self-harming behaviour are closely linked, and as many as 65–80% of people with BPD have engaged in some form of self-harming behaviour (Brickman et al., 2014). Furthermore, BPD is characterized by instability in interpersonal relationships, poor impulse control and low self-image (Gunderson et al., 2018). Repeated self-harming behaviour increases the risk of suicide, and mortality due to suicide is 10% (Ekdahl et al., 2011) which further creates a high burden for families with the risk of deteriorating their own life situations. Families’ ability to manage everyday life and maintain a stress-free environment is a good prognostic factor for recovery of people with BPD (Ekdahl et al., 2011). Families take the major role of responsibility and are the most important social support for people with BPD in society and thus need to take care of themselves in order to provide support for their family member with BPD (Kay et al., 2018). Living with a person suffering from BPD is described as being associated with stress, depression, grief and isolation; and family members express negative feelings towards their relatives and experience feelings of social humiliation, financial strain and marital discord (Kay et al., 2018). The majority of families of people with severe mental illness, regardless of type of mental illness, feel overwhelmed, unable to cope and exhausted with stress (Weimand et al., 2010). The burden experienced by families with a relative diagnosed with BPD has been noted to be even greater than those with other mental disorders (Bailey & Grenyer, 2013). Due to repeated self-harming behaviour many families fear that something devastating will happen to their loved one, making it difficult for them to prioritize their own well-being (Ekdahl et al., 2011). Previous research has also shown that family members of individuals diagnosed with BPD sometimes experience challenges and discrimination when attempting to engage with health services. They often feel devalued, unsupported and uninformed of the treatment process (Dunne & Rogers, 2013).

The first-line treatment for people with BPD includes some form of psychological intervention on an outpatient basis (Rameckers et al., 2021). Psychotherapeutic treatment has been used for quite some time and yet we know that patients with access to it are nevertheless high consumers of inpatient care, often for the purpose of suicide prevention. This may entail prolonged admissions where compulsory measures are also executed, which can worsen the condition and, in the long term, contribute to the person being deprived of their own ability and personal responsibility to handle difficult situations (Eckerström et al., 2020).

BA is a nursing-based crisis intervention designed to provide a timeout for patients in situations of increased stress to strengthen self-management in a safe environment and prevent self-harming behaviour (Eckerström et al., 2020). The idea behind BA is that a short-term self-managed hospitalization is possible for a maximum of 3 days per care occasion (Helleman et al., 2018). In case of increased stress or crisis, a break should be offered with crisis management during the care period. The admission is done on the basis of the patients’ desires without a doctor assessing whether there is a need or not. As previously mentioned, there are deficiencies in the content and structure of traditional inpatient care. Compared to traditional mental health services there is a lack of knowledge concerning how families of people with BPD experience BA. People with BPD as well as their families feel excluded and experience stigma and low quality of care within traditional mental health services (Barr et al., 2020). Families want to be involved and listened to, yet they report feeling obliged to pay an “emotional ransom”, which included feelings of being accused, being broken, being confused, and feeling lost (Lindgren et al., 2010). Families and their loved ones share a life. Although people’s life worlds often have several similarities with each other, there are always characteristics in a life world that make it completely unique (Dahlberg & Dahlberg, 2020). Therefore it is important for mental health professionals to listen to the lived experiences of both patients and their families (Dahlberg et al., 2008). By using life world research, nurses’ can increase their understanding of people by discovering, describing and clarifying their experiences (Dahlberg et al., 2008). The aim of this study was therefore to highlight experiences of being a family member of a person suffering from borderline personality disorder and self-harming behaviour with access to brief-admission.

## Method

### Design

To understand the lived experience among families of people suffering from borderline personality disorder and self-harming behaviour with access to BA, a phenomenological lifeworld perspective was chosen (Dahlberg et al., 2008). In the phenomenological lifeworld perspective people’s experiences are central and considered to be interacting with each other. The concept of life world is about the complex, subjective world where individuals live their lives and exist as interpretive beings (Dahlberg & Dahlberg, 2020). The life world perspective means trying to see, understand, describe and analyse the whole or parts of the life world as it is experienced (Dahlberg et al., 2008). Every human being is full of memories and previous experiences, which are meaningful. The phenomenological lifeworld perspective has been found useful to understand and make sense of family members’ lived experiences.

### Participants

Purposive sampling was used to recruit families of people with BPD and self-harming behaviour with access to BA in a region of southern Sweden. The patients decided which family member would be seen, and the families were asked to participate first after patients’ approval. Inclusion criteria were family members aged over 18 years. Family members who suffered from their own mental illness decided themselves whether they felt well enough to be able to participate in an interview, and no one declined for that reason.

### Data collection

The head of a psychiatric department in southern Sweden gave permission to conduct the study and mediated contact with health care staff working within BA. The staff helped identify patients assigned to BA. After the patients approval the family members were invited to participate in the study by phone. They received written information about the study and signed an informed consent form before entering the study. Place and time of the interview were decided by the family members to create precedents for a safe and relaxed interview environment for interviews. Five of the interviews were conducted in a secluded room at the psychiatric clinic, four via zoom and three thru telephone due to the covid-19 pandemic. The interviews were performed from November to December 2021 by third and last author, and recorded digitally. An interview guide was used to undertake interviews in an open-ended format and allowing the family members to speak freely about their individual lifeworld and experiences of BA (Dahlberg & Dahlberg, 2020). The interview guide was developed in close cooperation within the research team. The questions focused on the aim of the study: to describe the lived experiences of being a family member of someone with borderline personality disorder and self-harming behaviour with access to brief-admission. Furthermore the questions focused on whether, and if so how, BA has influenced their life-situation. To get in-depth interviews, probing questions were asked such as, “Can you tell me more?”, or further questions based on the informants’ previous answers. The first two interviews were performed as pilot interviews, and as no changes were made these were included in the analyses. The interviews lasted between 30 and 75 min. After the interview there was time for further reflection between the family member and the interviewer in order to address any troublesome feelings that may have arose during the interview. During the interviews notes were written down about expressions and emotions that appeared. These were used as an addition to the transcripts (Dahlberg & Dahlberg, 2020; Dahlberg et al., 2008).

### Data analysis

The goal of a phenomenological study is to describe a structure of meanings—of which the essential meaning is of essential importance (Dahlberg et al., 2008). First the transcripts were read repeatedly by all authors several times so they could become familiar with the text as a whole. Text which addressed the aim of the study was highlighted and sorted into meaning units. These meaning units were discussed within the research team until agreement was found on how to sort them into clusters. The text was then read separately by the authors to get a sense of the essential meaning and structures. At this stage, questions about family members’ lived experience, and if and how their life situations changed during BA guided the analyses to find a general structure that would illustrate the essence of the findings and its constituents. Constituents are variations and nuances of the family members’ lived experience. The core of the lived experiences was captured during the analysis and constituted the essence of the findings (Dahlberg & Dahlberg, 2020). During the analysis process the researcher must be curious about the unique experience of every family member, as all human beings have their own pre-understanding and understanding which leads us to understand and relate to the world in which we live (Dahlberg et al., 2008). Experiences and their meaning occur not from a human’s private inner world, but from relationships and the intersubjective world in which we share traditions, norms, and values with each other (Dahlberg & Dahlberg, 2020). Thus experience are a movement between the subjective experiences against the objective and a shared world. In the research process the researcher needs to bridle their preunderstandings so that unpredictable responses can be captured and to avoid misleading results. It is thus important to make sure our evolving understanding does not happen randomly or too fast, and that the understanding of the phenomenon needs to be questioned to open up many possibilities to reach full process of understanding (Dahlberg et al., 2008). The researchers bridle personal ideas and understandings with each other through the entire data analysis and consulted the raw data when necessary in order to avoid too much interpretation and to avoid the analysis being influenced by prior understandings (Dahlberg et al., 2008). By taking part of the phenomenon as described by the family members using a phenomenological lifeworld perspective, the essential meanings and the aim of the study were considered to be captured.

### Ethical considerations

The study was approved by the Ethical Review Board in in Linköping, Sweden, Dnr 2021–04567. All family members signed a written informed consent form after they were given verbal information about the aim and implementation of the study, the voluntary nature of their participation, and that they could withdraw at any time without giving a reason, (World Medical Association, 2013). All data were handled in accordance with the General Data Protection Regulation in Sweden. The research process and how the method and result are compiled have followed the COREQ guidelines (Tong et al., 2007).

## Result

A purposive sample of 12 family members was interviewed. The participants were aged between 22 and 75 years (mean age 46 years). Five participants were mothers, three sisters, three husbands and one was a close friend.

The results consist of a general structure that illustrates the essential meaning “essence” of the data as well as the more particular meanings “constituents” which illustrate the variations and nuances of the essence. The constituents are (1) Difficult feelings to live with, (2) The dream of a functioning everyday life, (3) BA creates security and (4) A betrayal by psychiatry.

### Essence

Families of people with BPD live with a constant fear and uncertainty about how their loved ones are feeling and whether they will injure themselves or die of suicide. Their whole life situation focuses on interpreting the feelings of their family member with BPD and trying to understand how their loved one feels. This sets their own lives aside which are put “on standby”. Living with constant worry and a need to control the loved one leaves no room for the informants to ever relax, no matter where they are. Even in situations where they are expected to have fun, this is made impossible as they are constantly reminded of the issue of their loved ones’ health, life and death. Feelings of guilt, shame and a bad conscience cause them to be dragged down into feelings of “darkness” where mental illness becomes a reality for them as well. They are constantly worried and overwhelmed by the pain of their loved one. When families gained access to BA it gave them hope for an improved life situation and was seen as a step closer to a functioning everyday life. It gave room for respite in which they could, at times, release anxiety and fear.

#### Difficult feelings to live with

Living with a loved one with BPD who engages in self-harming behaviour evokes a lot of emotions that can take over and control families’ lives. Emotions that are described as a constant worry and fear that the loved one will harm themselves or commit suicide mean that the families feel they can never really let go of control over what the relative is doing, and they feel a need to be constantly on hand and to monitor the current mood of their loved one.

I think if I compare with my colleagues [at work], I think I call or send many more messages in one day and check what my partner is doing (p 5)

Having recurring suicidal thoughts and suicide attempts made it difficult for parents to live with children who had BPD, and it emerged that parents carried their child’s pain. Viewing the child’s feelings of hopelessness took over the parents’ thoughts and made it difficult for them to concentrate on anything else. It appears that parents never feel better than the child feels at their worst, and they try to deal with feelings of helplessness and anger. At the same time, they do not want to show how they really feel in front of their children and therefore cry in secret when no one sees.

We call each other in the family; not everyone feels as bad all the time. An older sister can say that she is so angry with her right now, why can’t they just lock her up what is she doing, what has she done to herself, I am going insane! Then I can calm her down; next time it may be me who is angry. I can get very angry, she does not see what she has, she has her children, how can she do that to herself? (p 4)

Feelings of guilt and shame also occur within families, and they have a hard time allowing themselves to have fun. In these situations, they are reminded of thoughts and guilt about having forgotten their loved one. This leads them to prioritize their family member with BPD instead of their own well-being in order to be there for their loved one.

What if something happened during the time I had fun, how can you live with that? (p 5)

#### The dream of a functioning everyday life

Family members expressed that it felt like an effort to find a balance in everyday life where life can work and not just consist of basic routines. Life is described as a constant challenge to manage job, finances, children, school and activities. The family described uncertainty in not knowing when their loved one would return home during ordinary psychiatric care, which resulted in suffering for the whole family. Being able to take care of all the chores at home was stressful both for the family but also for the children who were sometimes picked up late from preschool and went to bed far too late. Access to BA made it easier to take care of the home and the children, and less time had to be spent travelling around and getting other family members to help. Family members further appreciated knowing that BA is time-limited.

The children did not know when their mother will return, will she come tomorrow or will she come home in August or October, November? … in the long run I think it is easier for the children if they notice that their mother is only away for two or three days, not for two or three months (p 2)

Access to BA also meant that more time could be devoted to children and other family members. It turned out that children can see if someone is unwell and can then withdraw and try to make themselves invisible, which was described as very worrying. As the loved one could apply for BA at an early stage, this could be prevented and there was more time to pay attention to the rest of the family and make the children feel secure.

It is difficult to make everyday life go together with children, job, finances; we feel trapped as a family when a family member can get sick very quickly and it paralyzes everything (p 2)

When their loved ones were given access to BA and had to take responsibility for seeking care themselves, it emerged that the care period became shorter and they were able to act in time and thereby avoided more severe periods of illness.

I think that this tool, BA, could help make everyday life work instead of the horrible existence that it sometimes is (p 2)

A network of people around was described as important for a functioning everyday life, particularly having people around who they could talk to when the challenges were at their worst. This network could consist of other family members, co-workers or friends.

You end up with your sister or with your mother or with a close friend. … Many of them close say: “Come to our house and have dinner so that the children can play with each other and then there will be a little break for you” (p 2)

Constantly prioritizing their loved one and ignoring their own well-being meant that many of the families suffered from their own mental illness and fatigue diagnoses, which in turn could lead to them taking sick leave. It appeared difficult for the families to concentrate on their own recovery, and there was a need to discuss their life situation with a professional nurse, counsellor or psychologist.

Every two weeks I see a nurse at the psychiatric clinic, just to cope … and I have a sewing room at my home with my sewing machines and fabrics where I can rest … either I sew something or I sit there and just stare, depending on how tough it is (p 9)

#### BA creates security

The families reported feeling that when their loved one gained access to BA, it gave them a feeling of calm, and a hope that their life situation, for themselves and their loved ones, could be safer. They felt reassured to know that help was only a call away; it was not as dramatic as previous hospitalizations when both the police and ambulance had to be contacted. A sense of security arose in the fact that their loved one did not have to seek care via the psychiatric emergency room, which could sometimes take several hours. It further emerged that family members felt safe to know that their loved one received help without having to fight to be hospitalized, because the doctor judged that there was no need or because all inpatient places were occupied. It also provided security when a BA stay was linked to a specific department, which is not the case in ordinary care.

Care will be very different depending on which department has a place, just at that time … to be able to know instead, that if you apply to BA, well then it is this department and this staff, it feels reassuring (p 5)

The knowledge that their loved one has access to BA and help at an early stage before a self-harming behaviour or suicide attempt has occurred also provided security. When their loved one was allowed to enter BA for a few days, worsening psychiatric symptoms expressed as darkness and chaos were usually avoided, which usually affected the family, and BA was believed to slow the course of the disease. The disease had previously progressed so far that inpatient care was required. Self-harming behaviour increased in connection with their relatives being hospitalized, which had caused a sense of great insecurity.

It is a security with BA for me as a relative; she does not have to be admitted through compulsory care. When she is more or less stuck in the ward, it affects life in a completely different way, the children are not allowed to see her (p 8)

When their loved ones were admitted to BA, they could relax and for the moment feel peace when they knew they were in a safe place. Having access to BA and knowing that their loved ones were there created a calm feeling and an awareness that professionals took over the responsibility of care and provided supervision. The security of having their loved one under supervision and the knowledge that he or she was not locked in meant that they could relax.

I think it is a great relief with BA, because it is a huge fear, what she will come up with, even if she is not suicidal, you are still worried because you never know when it will tip over (p 9)

#### A betrayal by psychiatry

When family members’ loved one gained access to BA, it gave hope for a safer life; however, when they were instead denied BA admissions several times because the place was occupied, it was perceived as a betrayal by the care providers and contributed to the family losing confidence in psychiatry. Patients withdraw from calling to seek a BA place again for fear of being denied. The joy and relief that they previously felt turned into feelings of frustration, disappointment, and despair, and not least, an insecurity among the families.

It has worked worse than we thought … we looked forward to it when it was offered … it sounded good to be able to come in a little bit earlier than when it is urgent … . but what happened was that when we called there was no place available (p 5)

In the event of a denied place at BA, the loved ones risk a worsening condition, which can lead to emergency admissions in usual care in which families did not feel they were involved. Anxiety was evoked among the families when they could not call and ask for a BA place themselves. During emergency admissions in conventional care, doctors at the psychiatric emergency room may propose a new drug treatment without consulting or discussing with families or regular doctors. Families reported feeling criticized for being too involved in the lives of their loved one, which created uncertainty and made them feel that they were either too little or too much involved. The families’ life situation was severely affected when their loved ones were admitted to emergency care. It was a great disappointment not to be allowed to participate or to receive feedback.

When the person comes home, then it is me who takes care of that person … You are cared for a period in hospital when it is at its worst, but I care very often … then to not get any feedback … (p 2)

One informant described a Christmas eve before BA when their relative was allowed to come home only in the afternoon and after many hours of persuasion. This was experienced as a disaster for the family. It also emerged that psychiatry is perceived as a closed world where no one cares about the informants’ perceptions, which in itself creates anxiety, despair and frustration among them.

There have been times when I have called to ask how she is feeling and I do not find out anything … as a relative it is so hard (p 4)

After BA these barriers were slowly removed.

## DISCUSSION

### Methodological considerations

The research method using a phenomenological life world approach was chosen to understand and describe the families’ life worlds (Dahlberg et al., 2008). The method assumes that people try to give everything a meaning and significance. The researchers do not search for the right answers but try to understand and create meaning around the families’ experience of their life world, which is stated in the essence. The understanding of family members’ life situations would not be possible without the phenomenological life world perspective as families and their loved ones have an immediate relationship to share with others (Dahlberg & Dahlberg, 2020). One limitation may be that the interviews were conducted by two people and their interview techniques may have differed. To minimize any associated risks, the interview questions were developed by all the authors together, and during the interviews and the analysis the authors discussed the interview text in order to understand and interpret it equally. The sample consisted of eight women and four men. That so few men participated may be due to the fact that it was the patients, of whom all were female, who themselves selected the family members. Perhaps they would rather seek out female family members to talk to about their feelings and moods. In any case, the families varied in age and how they were related and how involved they were in BA, which in itself can increase the possibility of capturing varying perceptions and thus increase credibility of the study (Polit & Beck, 2021).

Due to the ongoing Covid-19 pandemic and its high spread of infection, most interviews were conducted digitally via Zoom or telephone. A weakness with telephone interviews may be that researchers miss what is said between the lines and read via body language. Further small talk and follow-up questions become more natural in an interview situation face to face (Polit & Beck, 2021). The telephone interviews were nevertheless perceived as rich in content, and did not differ in scope from those conducted face-to-face. Interviewing family members of people suffering from BPD and self-harming behaviour evoke ethical aspects that the researchers have reflected upon as the researchers are responsible for providing support to participants (Dahlberg et al., 2008). Even though the families expressed an appreciation in being able to share their story we noticed that the family members continually returned to talking about the patient’s life situation, and the interviewers repeatedly had to remind them about that it is their life situations that are of interest. This can be understood based on the suffering that emerges during many of the interviews and the fact that families are known to put themselves aside as they largely take on the role of caregiver and deal with the emotional burden of their family member without any education or support (Dunne & Rogers, 2013; Hoffman et al., 2007). It can further be understood in the context that relatives traditionally have not been included and listened to in psychiatric care (Lindgren et al., 2010).

To strengthen the trustworthiness of the essence and its constituents, the results are presented together with quotes (Dahlberg, 2008). When conducting phenomenological open-ended interview studies it is not the number of people interviewed that is of importance but the richness and depth of the interviews (Polit & Beck, 2021). The interviews in this study were rich in descriptions, and during the analysis, content in the different interviews were repeated which enhances the trustworthiness of the findings. Transferability in qualitative studies is determined by whether the results are transferable to a similar context (Polit & Beck, 2021). To help the reader decide if the results are transferable, a clear description is given of the environment in which the study was performed including BA, the demographic background of participants and the study process.

### Discussion of results

The families’ entire life situations are governed by strong feelings about their loved ones’ mood. Constantly having to monitor their loved one so that they do not hurt themselves, puts the families own lives on standby and the children’s routines are disturbed. When their loved ones gained access to BA, it gave them hope of an improved life situation and of being a step closer to a functioning everyday life. It gave room for respite, which meant that at times the families could let go of anxiety and fear. When the loved ones were denied BA, it felt like a betrayal that contributed to the families losing confidence in psychiatry.

One of the most important results in this study shows that the families’ life situations are characterized by anxiety and fear about how their loved ones feel and they experience uncertainty about what tomorrow will look like. Although there are no previous studies that show how families of the loved one with access to BA experience their life situations, it has emerged that the burden experienced by families of someone diagnosed with BPD involves greater suffering than for those with other mental disorders (Bailey & Grenyer, 2013), and carries a lot of fear and anxiety (Hoffman, 2007). It can be rewarding for the family to provide support and care but it also places a significant burden on them which can cause suffering. Health professionals taking part of the families’ experiences and life world, improves their understanding of their situation (Dahlberg et al., 2008) and are better equipped regarding how to support the whole family towards better health. Families of people with BPD are in a vulnerable position, where stressful situations over time can jeopardize their mental well-being and quality of life (Bailey & Grenyer, 2013). In this study, feelings of helplessness and anger and a constant need to monitoring their loved one emerged so that he or she did not get hurt, which had led to several of the family members themselves suffering from fatigue, depression, sadness, pain and anxiety. It also emerged that their own lives had to be on standby. In order for the family to be able to manage their life situation and for their health to improve, they need support that can be offered in support groups (Hoffman et al., 2007; Kay et al., 2018). Another possible arrangement would be to offer the families with loved ones receiving BA some form of family support where they can share their feelings and lived experience with health care staff or others in similar situations and feel listened to.

Another finding in this study was that the whole family suffered when the loved one became ill. The families who had children described how they had previously been worried about the uncertainty of when the parent would return home. It also emerged that routines were disturbed, and the children did not go to bed on time in the evenings. When a parent suffers from mental illness, the children become carriers of their anxiety (Dam & Hall, 2016; Dam et al., 2018). They may experience a lack and longing for the parent and worry about being placed in a foster home or that the parent will commit suicide. In this study, it also emerged that other children in the family tried to hide away when a sibling got ill. Previous studies have shown that some children do not show any emotions at all but keep to themselves (Dam & Hall, 2016), while other children experience the mental illness of their parents as a heavy burden and find it difficult to be left alone with these thoughts and feelings (Dam et al., 2018). This constant worry can lead to children not playing with their friends because they feel guilt, shame, sadness or are ashamed of their parent. It is important to consider the families’ experiences over their life situation as their life worlds are linked to their loved ones (Dahlberg et al., 2008). Understanding each family’s unique situation is a prerequisite to supporting the whole family towards better health. Children of parents with mental illness can take a great responsible for their ill parents (Dam & Hall, 2016). Having knowledge of mental illness makes it easier for children to manage their situation, which in itself leads to them isolating themselves to a lesser extent (Dam & Hall, 2016). Based on this, it is important to create conditions for the children to feel safe and let them know that when the parent applies for a BA it will be a maximum of 3 days before he or she returns home. Dam et al. (2018) emphasize that emergency rooms in psychiatric inpatient care create a stressful situation for children. It is important to create a safe life for the children, otherwise there is a risk that they will grow up and suffer from their own mental illness (Dam et al., 2018). Among the families in this study, it emerged that with access to BA, the loved ones were given the opportunity to maintain a more normal life situation and a functioning everyday life where the children could be offered a safer life. This is an important finding also from the patients’ perspectives as people with BPD may have difficulty with the parent—child relationship and place children at increased risk of negative outcomes (Florange & Herpertz, 2019). Thus, BA as a nursing intervention can help create conditions for maintaining a better relationship between the children and the parents as well as other family members. In this study, it emerged that the families felt that the knowledge of rapid hospitalizations provided security. This has previously been observed in patients with access to BA (Helleman et al., 2018). However, family members in this study describe that they live with a constant monitoring to avoid their loved one harming her/himself, and that a deterioration can happen quickly. They live in circumstances where the loved one can quickly switch between extremes in emotional states (Gundersen et al., 2018). For the families in this study, it emerged that BA gave them the opportunity to breathe a sigh of relief and let go in the knowledge that their loved one was in safe hands during BA. Still, they had difficulties in letting go of the loved one based on previous experiences of traditional care when self-harming actions had been carried out, which led them to feel the need to ensure that BA is a safe place. The Swedish regulation, Health and Medical Services Act (SFS 2017: 30) emphasizes the importance that the care offered should be safe and that families are involved as much as possible. Families in this study expressed a desire to be more involved than they were. Letting the family be involved in their loved one’s care is a fundamental foundation from a phenomenological lifeworld perspective of good care, as the families’ and their loved ones’ lived experiences are considered to be linked and interacting with each other (Dahlberg & Dahlberg, 2020). It has been seen before that families who are not offered the opportunity to participate in their loved ones’ care feel invisible, not listened to and excluded from care (Dunne & Rogers, 2013; Kay et al., 2018). BA, in its current approach, does not involve the family (Helleman et al., 2018). Based on the families’ statements in this study, a possible future proposal may be to ask relatives when BA commences, if, and if so how, they want to be involved.

It is also important to ask about the families’ need for support and to support them so that they in turn can handle stress and daily chores, which are the prerequisites for them to be able to support their loved one towards health and recovery (Ekdahl et al., 2011). However, there are challenges in involving families in psychiatric inpatient care in circumstances where care staff are prevented from involving them due to confidentiality (SFS 2017:30). Confidentiality aims to protect the patient’s integrity where the reverse side is when the patient use their right to confidentiality and thereby exclude the family. This leads to the families losing confidence in the care system (Dunne & Rogers, 2013; Hoffman et al., 2007; Kay et al., 2018). It is important that the patient’s integrity is taken into account. However, it must be taken into account that the family takes responsibility when their loved one is not admitted to psychiatric inpatient care. During BA, which is limited in time, there are no restrictions on patients’ movement and they are allowed to come and go from the ward as they want. This provides better conditions for the whole family to not be separated during long care periods, even though the loved ones request confidentiality. Psychiatric care has a history of excluding relatives and blaming them for having caused mental illness in the patient (Lawn & McMahon, 2015). This lack of participation has been linked to mental illness in the family (Ostlund & Persson, 2014). It is further shown that families who are involved in health care to a greater extent, increase their own well-being, and have a better opportunity to receive information about the disease, which helps them to set boundaries and prioritize their own health. The family must be recognized as a partner, as there are great benefits for all parties if all the family members’ experiences are revealed as they share memories, norms and a life with one other (Dahlberg & Dahlberg, 2020). BA is a relatively new nursing intervention that has the potential to give families the conditions to get involved with their loved one’s care in the psychiatric context.

### Conclusion and implications

The families’ entire life situations were governed by strong feelings and constantly having to control their loved ones not to put themselves in danger. The phenomenological life-world perspective made it possible to capture experiences of BA as inspiring hope for a brighter future and helpful for families to let go of anxiety and fear. BA also created prerequisites for strengthening the relationship with their children as well as other family members who are in a stressful and vulnerable position. However, denial of access to BA due to restrictions in ward places awoke feelings of betrayal and frustration, and families requested that they wanted to be more involved. Taking into account both the families’ and their loved ones’ life situations, BA must be available when needed; and due to the responsibility and daily monitoring families take on make them vulnerable and in need of support. The understanding of the families’ unique life-worlds highlight the families’ need of sharing strong feelings and being listened to in order to support their loved ones towards health. This was an underlying issue for the well-being of the whole family.

## Data Availability

Data available on request due to privacy/ethical restrictions.
